# Association between visual field damage and corneal structural parameters

**DOI:** 10.1038/s41598-021-90298-0

**Published:** 2021-05-24

**Authors:** Alexandru Lavric, Valentin Popa, Hidenori Takahashi, Rossen M. Hazarbassanov, Siamak Yousefi

**Affiliations:** 1 Computers, Electronics and Automation Department, Stefan Cel Mare University of Suceava, Strada Universității 13, 720229 Suceava, Romania; 2 Department of Ophthalmology, Jichi Medical University, Tochigi, Japan; 3 Department of Ophthalmology and Visual Sciences, Paulista Medical School, Federal University of São Paulo, São Paulo, Brazil; 4 Department of Ophthalmology, University of Tennessee Health Science Center, Memphis, TN USA; 5 Department of Genetics, Genomics, and Informatics, University of Tennessee Health Science Center, Memphis, TN USA

**Keywords:** Medical research, Preclinical research

## Abstract

The main goal of this study is to identify the association between corneal shape, elevation, and thickness parameters and visual field damage using machine learning. A total of 676 eyes from 568 patients from the Jichi Medical University in Japan were included in this study. Corneal topography, pachymetry, and elevation images were obtained using anterior segment optical coherence tomography (OCT) and visual field tests were collected using standard automated perimetry with 24-2 Swedish Interactive Threshold Algorithm. The association between corneal structural parameters and visual field damage was investigated using machine learning and evaluated through tenfold cross-validation of the area under the receiver operating characteristic curves (AUC). The average mean deviation was − 8.0 dB and the average central corneal thickness (CCT) was 513.1 µm. Using ensemble machine learning bagged trees classifiers, we detected visual field abnormality from corneal parameters with an AUC of 0.83. Using a tree-based machine learning classifier, we detected four visual field severity levels from corneal parameters with an AUC of 0.74. Although CCT and corneal hysteresis have long been accepted as predictors of glaucoma development and future visual field loss, corneal shape and elevation parameters may also predict glaucoma-induced visual functional loss.

## Introduction

While intraocular pressure (IOP), age, disc hemorrhage, and optic cup characteristics have been long identified as classic risk factors for development of primary open-angle glaucoma (POAG)^1,2^, the Ocular Hypertension Treatment Study (OHTS) suggested central corneal thickness (CCT) as a new risk factor for development of POAG^3^. Since then, several other studies confirmed that thin CCT may predict glaucoma development and future vision loss^4,5^.

A significant segment of literature now suggests that corneal biomechanical^6,7^ parameters may be associated with glaucoma^8–11^. Corneal hysteresis (CH)^12^, which reflects a viscous property of the cornea, has been shown to be a significant predictor of glaucoma development^13^. Congdon and colleagues^13^ first showed that CCT and CH are both associated with development of POAG. This new finding was further confirmed by several follow up studies^14–17^. A recent study suggests that CH may predict visual field progression in suspected eyes with apparently well-controlled IOP^18^.

The role of CCT is clinically important because it affects IOP measurements which can be misleading in glaucoma assessment^17,19,20^. The problem is even more critical, since a significant number of myopic patients in the US undergo refractive surgeries that affect IOP^21,22^. As such, models that explain the association between corneal anatomy and glaucoma risk independently from IOP are unmet needs.

Compelling evidence confirms glaucoma-induced progression of visual field loss can be delayed or prevented by reducing IOP^3,4,23–26^. Since glaucoma-induced visual field loss is irreversible, it is essential to predict visual field progression to prevent future vision loss through earlier intervention including reducing IOP^27^. Therefore, methods that can predict visual field damage are highly desirable in clinical practice.

Previous studies have shown the role of CCT and CH in development of POAG^3,28–32^.

Recently, several teams have attempted to estimate visual field parameters from optical coherence tomography (OCT) measurements using conventional statistical approaches^33–35^ or emerging deep learning models^36–38^. Predicting one retinal factor (VF severity level here) from another retinal factor (retinal structure captured by OCT here) might not be considered surprising. However, no study has yet investigated the independent role of corneal topography, pachymetry, and elevation on POAG development.

The purpose of the current study was therefore to investigate whether there is an association between corneal structural parameters and the severity of visual field damage using a retrospective dataset including both corneal and visual field measurements.

## Methods

### Subjects and datasets

This study was performed in accordance with the ethical standards in the Declaration of Helsinki and institutional review board (IRB) was submitted and approved by the “Jichi Medical University IRB Office” and informed consent was obtained from participants. The data was de-identified in Japan before any further processing and data use agreements were signed among parties to use data. Corneal parameters were collected from 676 eyes of 568 patients using anterior segment swept source optical coherence tomography (SS-OCT) instrument (CASIA, Tomey, Japan). In the anterior segment mode, each 3-D image consists of 128 B-scans (cross-sectional images) and 512 A-scans. In the corneal-map mode, each 3-D image contains 16 B-scans and 512 A-scans. The Topo-Pachy-Map scan protocol was used, which is composed of 16 evenly-spaced radial B scans. The total scan duration was 0.3 s for measurement of corneal thickness and corneal topography. Visual fields were collected by Standard automated perimetry (SAP) tests using the Swedish Interactive Thresholding Algorithm (SITA) and 24-2 strategy on the Humphrey Field Analyzer II-i (Carl Zeiss Meditec, Inc, Dublin, California, USA) from the same eyes. The inclusion criteria were: (1) the time interval between corneal data collection and visual field testing was less than 12 months for all subjects, (2) subjects that did not present any other ocular or systemic disease that could affect the optic nerve or visual field, (3) visual fields with ≤ 33% fixation losses or ≤ 20% false-positive errors, and corneal images meeting CASIA reliability metrics. Corneal specialists (including Hidenori Takahashi) clinically determined testing requirement for patients. Visual field testing was collected from patients with glaucoma or suspect of being glaucoma. However, there may be eyes in our study with conditions such as cataract, keratoconus, or visual acuity problems.

Visual fields were assessed based on SAP software-provided glaucoma hemifield test (GHT) and pattern standard deviation (PSD). Visual fields were considered abnormal if GHT was outside of normal limits or PSD was outside of 95% normative range (*P* < 0.05)^39,40^. Abnormal visual fields were also stratified to three severity levels of early, moderate, and severe visual field damage, based on mean deviation (MD) measurements that were better than − 6 dB, between − 6 and − 12 dB, or worse than − 12 dB, respectively.

### Machine learning analysis

**Figure 1:**
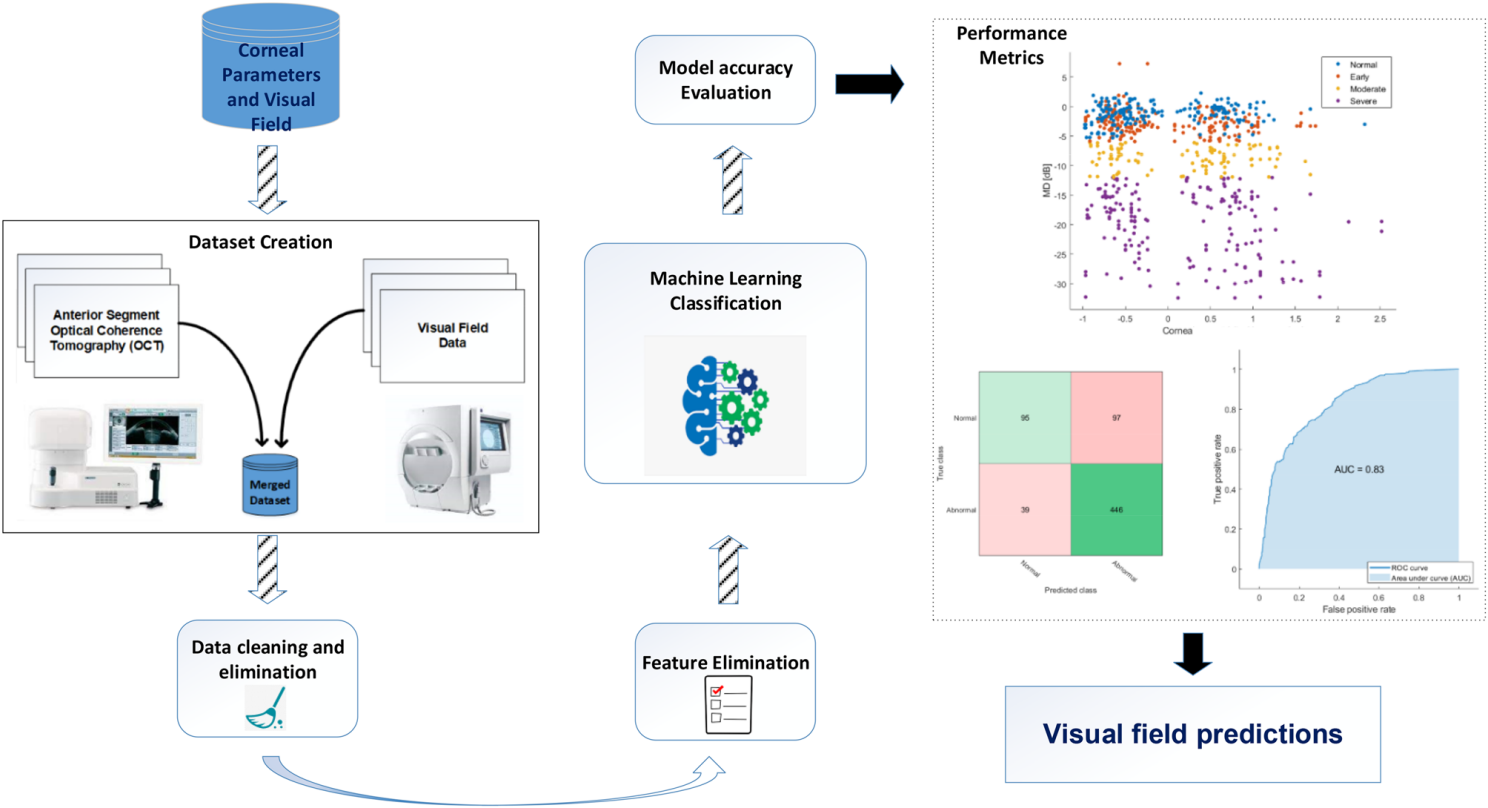
The pipeline of the proposed machine learning approach.

Figure 1 shows the diagram of the pipeline that was developed to investigate the association between corneal parameters and visual field damage. More specifically, several machine learning models were employed to detect visual field damage from corneal parameters only. The problems were framed as two-class (normal versus abnormal visual field) and four-class (normal, early, moderate, and severe visual field damage) classification tasks and asked whether the machine learning classifiers can detect visual field abnormality and visual field severity level from corneal parameters only. All corneal parameters were fed into the machine learning models with visual field damage and severity levels as outcome labels.

### Subset of corneal parameters predicting visual field damage

The elevation of the anterior and posterior cornea surface is represented by vertical and horizontal axes offset, vertical and horizontal axes real indices, and best fit sphere. The CASIA SS-OCT essentially assesses the elevation data and the reference shapes through commonly used parameters such as elevation, radius in horizontal and vertical, corresponding shape factors in horizontal and vertical directions, and corneal position parameters such as translational displacements in three directions and rotational displacements in three angles. To this end, best-fit-sphere (BFS), best-fit ellipse, and best-fit-toric ellipsoid are used. In fact, anterior and posterior elevation values are compared against the thinnest pachymetric points found in the BFS elevation maps within the 5 mm central zone as a reference surface sphere. Normal anterior BFS is typically less than 10 μm while a normal posterior BFS is usually less than 15 μm.

Corneal shape is also described by the conic shape parameters. Corneal eccentricity (ECC) indicates the departure of the peripheral curvature from the apical radius and so defines the degree of asphericity. Corneal surfaces may be spherical, prolate aspheric (steeper at the center and flatter at the periphery), oblate aspheric (flatter at the center and steeper at the periphery), elliptical, parabolic, or hyperbolic. Assessing the corneal changes in 4 and 12 mm zones reflects ECC and determine the values of asphericity necessary to maintain the physiological value of the corneal spherical aberration. Base Curve (BC) in vertical or horizontal directions reflects the thinnest point of the cornea. Spherical aberration is a rotationally symmetric aberration in which the light rays pass through the paraxial zone of the pupil focus at a different distance than the rays that pass through the marginal pupil.

Similar to multivariate statistical models, machine learning models are also less effective when a large number of predictors are fed into the model. As such, the most promising corneal parameters for detecting visual field damage were identified. More specifically, a framework was developed to evaluate the power of different combinations of corneal parameters in detecting visual field damage. A feature subset selection^41^ approach was employed to evaluate the worth of a subset of corneal parameters by considering the individual detection ability of each corneal parameters (similar to a statistical univariate model) along with the degree of redundancy among all parameters (similar to a statistical multivariate model).

More specifically, we generated different subsets of corneal parameters and assessed the worth of subsets in detecting visual field damage. We started with an empty subset and gradually added corneal parameters by searching all corneal parameters using greedy hill climbing steps augmented with a backtracking facility^41^. We stopped the process when no further improvement in accuracy was observed. Feature selection essentially provides several advantages such as improved visualization, enhanced understanding of input data, lower computational complexity, avoidance of overfitting, and improved accuracy^42^.

Machine learning models were also independently employed to detect visual field damage and severity levels of eyes using only this subset of corneal parameters and tested based on tenfold cross validation of the AUC and accuracy. Feature selection algorithm was performed in WEKA^43^ and all machine learning models were implemented in Matlab^44^.

Matlab is an integrated framework that allows design and development of many engineering systems including machine learning algorithms. Users can train and different machine learning models such as ensemble machine learning bagged trees classifiers that we have employed in this study. Models can be evaluated using several accuracy metrics such as area under the ROC curves.

## Results

This study included 676 eyes from 568 with both corneal measurements and SAP visual field testing through HFA. Table 1 presents the demographic and clinical factors of the cohort in this study. Mean age was 61.2 years, average MD was − 8.0 dB, and average CCT was 513.13 um.

**Table 1:** Study characteristics.

Selected parameter	Mean ± standard deviation (SD)	Range
Age (year)	61.2 ± 19.9	10–88
MD (dB)	− 8.0 ± 8.5	− 32.4 to 7.3
PSD (dB)	5.83 ± 4.2	1.01–16.96
CCT (µm)	513.13 ± 53	336–652

*MD* mean deviation, *PSD* pattern standard deviation, *CCT* central corneal thickness, Values are presented as mean ± standard deviation.

Table 2 represents the characteristics of the eyes in terms of visual field damage. A total of 192 eyes had normal visual fields while 484 eyes had abnormal visual fields based on GHT and PSD.

**Table 2:** Eyes with and without visual field damage.

Criteria	Visual field status	Number of eyes
GHT within normal limits and PSD with *P* ≥ 0.05	Normal	192
GHT outside of normal limits or PSD with *P* < 0.05	Abnormal	484

**Figure 2:**
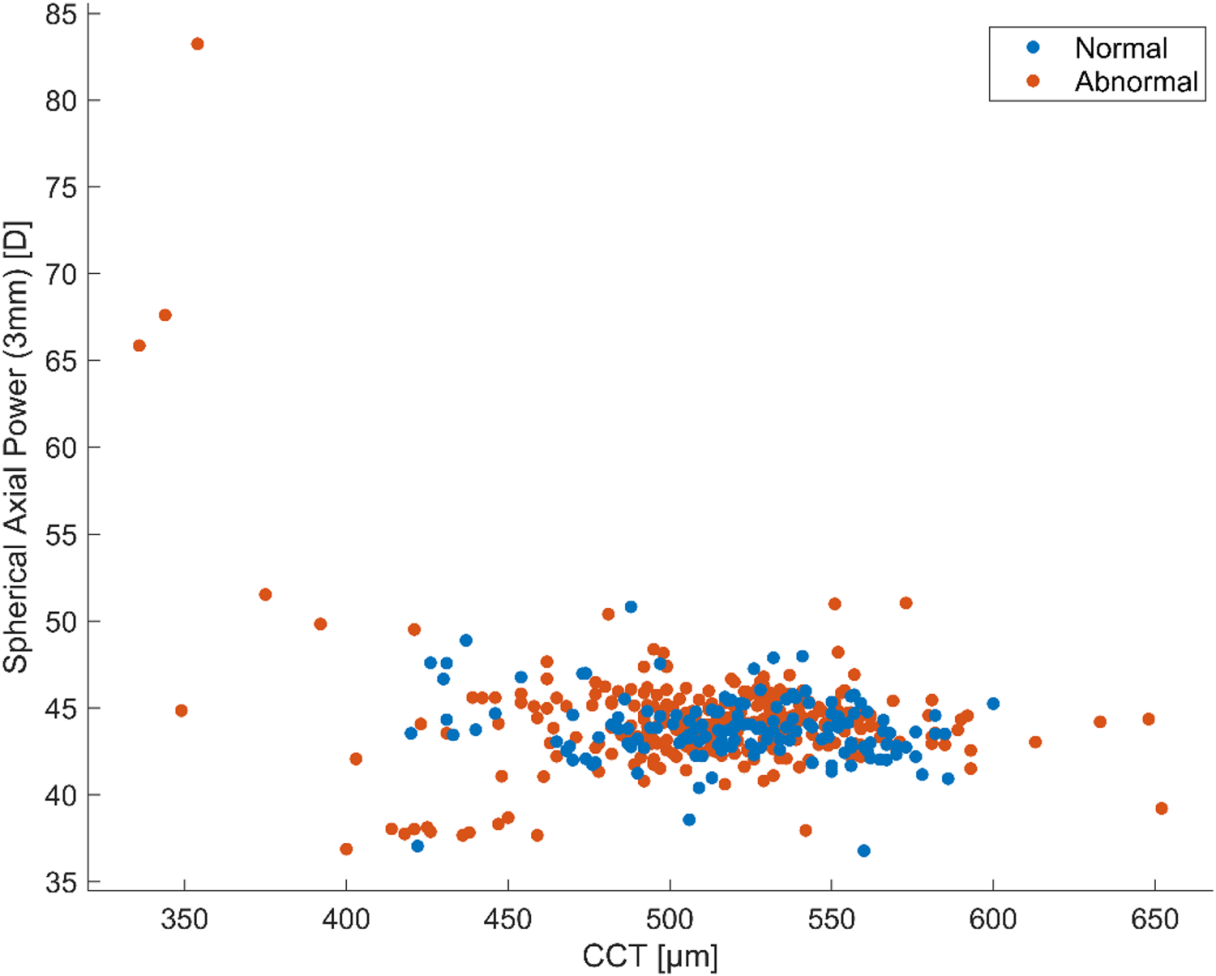
Class distribution of central corneal thickness (CCT) and spherical axial power of normal eyes and eyes with visual field damage.

Figure 2 presents the distribution of the CCT versus spherical axial power in 3 mm zone of normal eyes and eyes with visual field damage. As can be seen, not all corneal parameters are discriminative for the eyes in these two groups.

**Figure 3:**
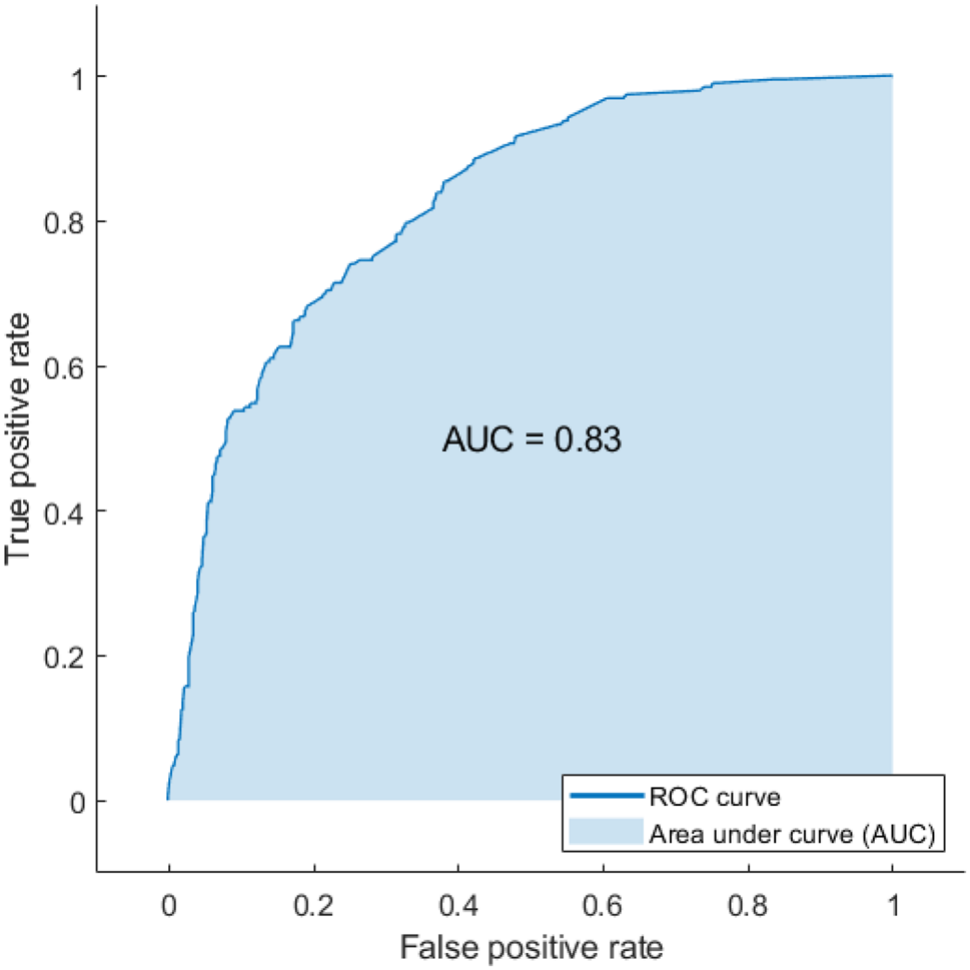
ROC curve of the subspace of ensemble machine learning bagged trees model for detecting visual field damage (normal versus abnormal) from corneal parameters.

**Figure 4:**
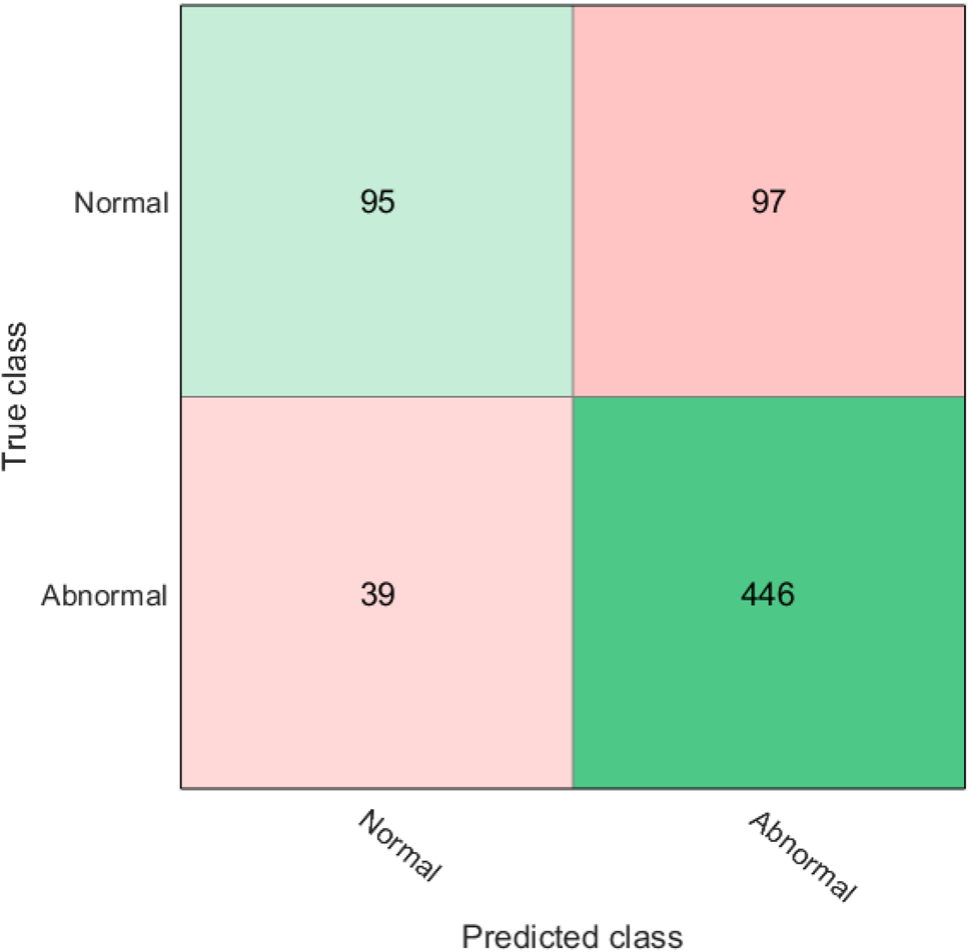
The true versus predicted visual field damage obtained using the ensembles machine learning bagged trees model.

The ROC curve for detecting visual field damage from corneal parameters is shown in Fig. 3. The predicted visual field damage from corneal parameters versus the true visual field damage of eyes at different severity levels is presented in Fig. 4. The best performing machine learning model to detect visual field damage in this subset was a subspace of ensemble machine learning bagged trees model that achieved an AUC of 0.83 and an overall accuracy of about 80%.

Table 3 lists the number of eyes with different visual field severity levels. A total of 192 eyes had mild visual field damage, while 107 and 185 had moderate and severe visual field damage, respectively.

**Table 3:** Number of eyes with different visual field severity levels.

Criteria	Visual field severity level	Number of eyes
GHT and PSD in normal regions	Normal	192
MD $\ge \hspace{0.17em}-\hspace{0.17em}$6 dB	Early	192
− 12 dB $<\hspace{0.17em}$MD $\le \hspace{0.17em}-\hspace{0.17em}$6 dB	Moderate	107
MD $<\hspace{0.17em}$− 12 dB	Severe	185

**Figure 5:**
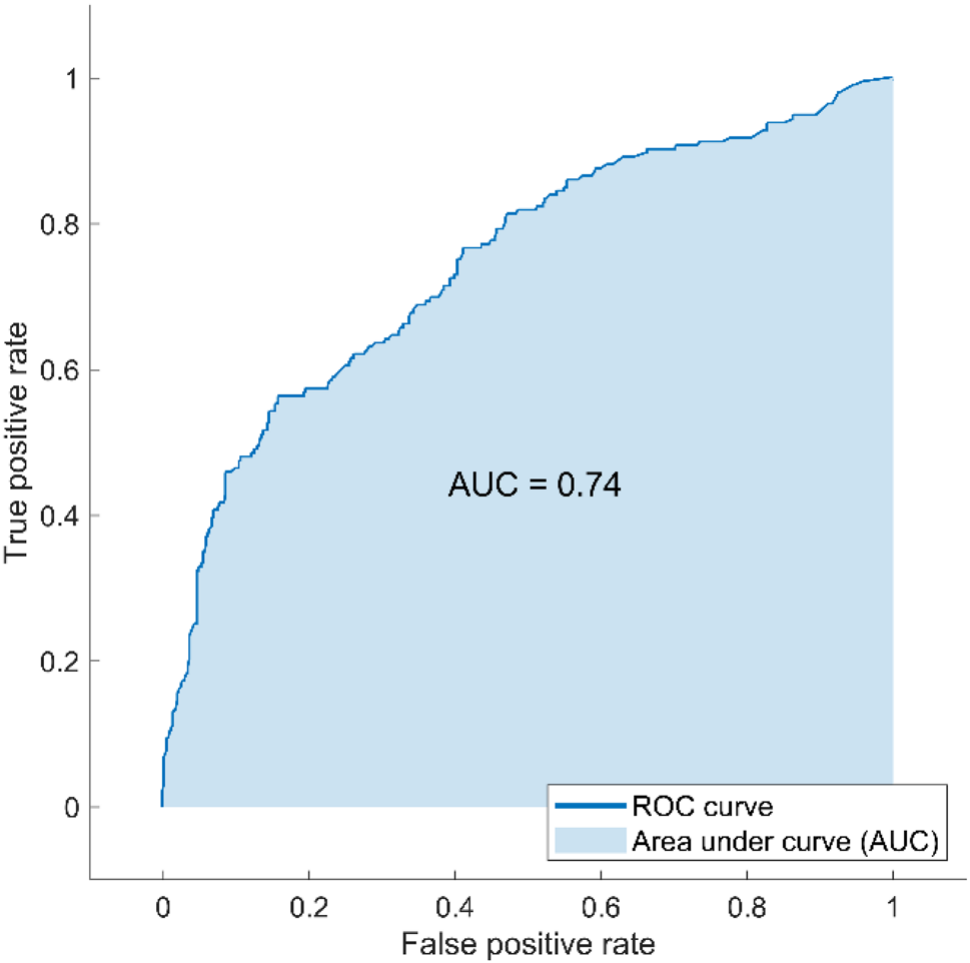
Receiver operating characteristic (ROC) curve of ensemble machine learning bagged trees model for detecting four visual field severity levels including normal, early, moderate, and severe.

The best preforming machine learning model to detect different visual field severity levels was tree-based machine learning, which achieved an AUC of 0.74 and an overall accuracy of approximative 60%. The ROC curve for detecting different visual field severity levels from corneal parameters is shown in Fig. 5.

**Figure 6:**
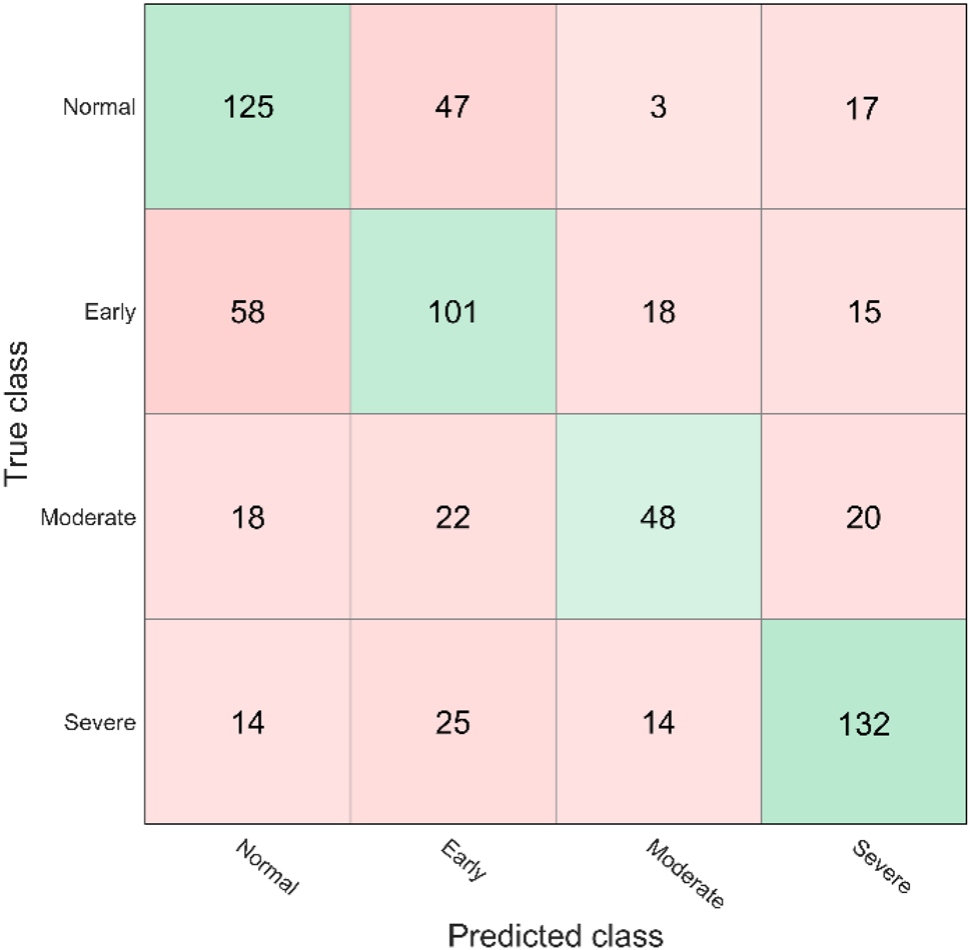
The predicted visual field damage from corneal parameters versus the true visual field damage of eyes at different visual field severity levels.

The predicted visual field severity level from corneal parameters versus the actual visual field severity level is presented in Fig. 6.

Table 4 lists 12 most promising corneal parameters that were identified using feature subset selection. 7, 2, 2 and 1 parameters belong to topography, elevation, pachymetry and keratometry, respectively. The table also includes ranking of the features based on Pearson correlation which evaluates the correlation of each selected feature with the output visual severity class.

**Table 4:** Selected features from optical coherence tomography (OCT) imaging of cornea.

Selected feature (unit)	Description	Importance rank	Measurement
Corneal eccentricity (4 mm region)	Height anterior index eccentricity (ECC) at 4 mm region	0.18	Topography
Vertical axis offset difference Y axis (mm)	Offset difference (vertical axis offset Y)	0.13	Topography
Base curve (BC) Y axis (mm)	Vertical axis of the BC measured using enhanced BFS	0.11	Topography
Elevation anterior index of the best fit sphere (BFS) Y axis (mm)	Y axis of the elevation anterior index of BFS	0.11	Elevation
Pupillary offset (PO) (mm)	Pupillary offset in mm	0.09	Pachymetry
Horizontal axial index (mm)	Horizontal axial index of total anterior and posterior segments	0.06	Topography
Spherical aberration coefficient in 4 mm (um)	Spherical aberration coefficient of the anterior segment in 4 mm zone	− 0.06	Keratometry
Corneal eccentricity (15 mm region)	Height anterior index eccentricity (ECC) at 15 mm region	− 0.09	Topography
Corneal eccentricity (12 mm region)	Height anterior index eccentricity (ECC) at 12 mm region	− 0.09	Topography
Elevation anterior index of the best fit sphere (BFS) Z axis (mm)	Vertical axis of the elevation anterior index of BFS	− 0.11	Elevation
Offset X axis anterior steep area (mm)	Horizontal axis of the anterior steep area	− 0.13	Topography
Best fit sphere (BFS) Z axis (1 mm region) (mm)	Vertical axis of the anterior best fit sphere (BFS) in the 1 mm region	− 0.20	Pachymetry

**Figure 7:**
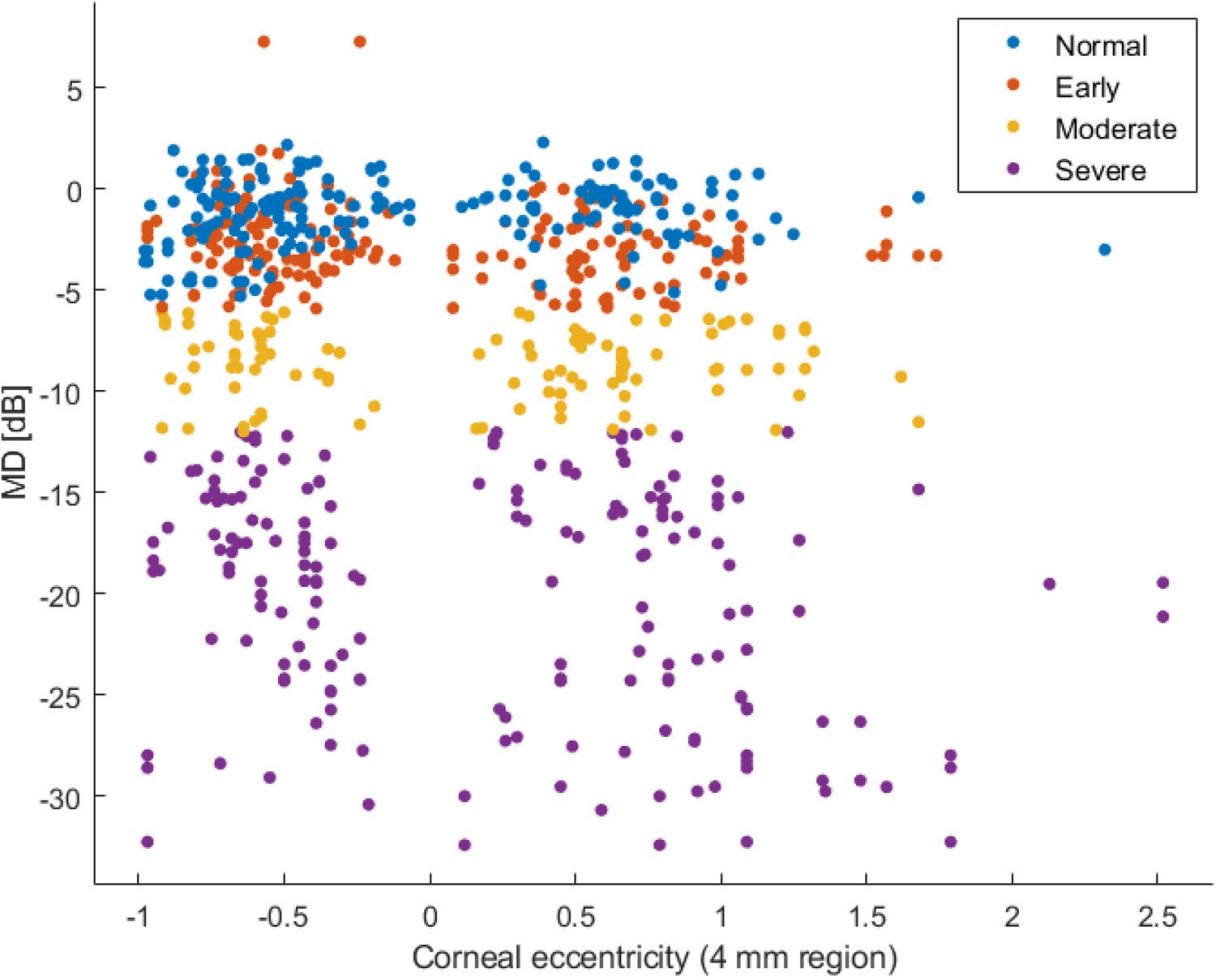
Classes distribution of corneal eccentricity in 4 mm region and the mean deviation (MD).

**Figure 8:**
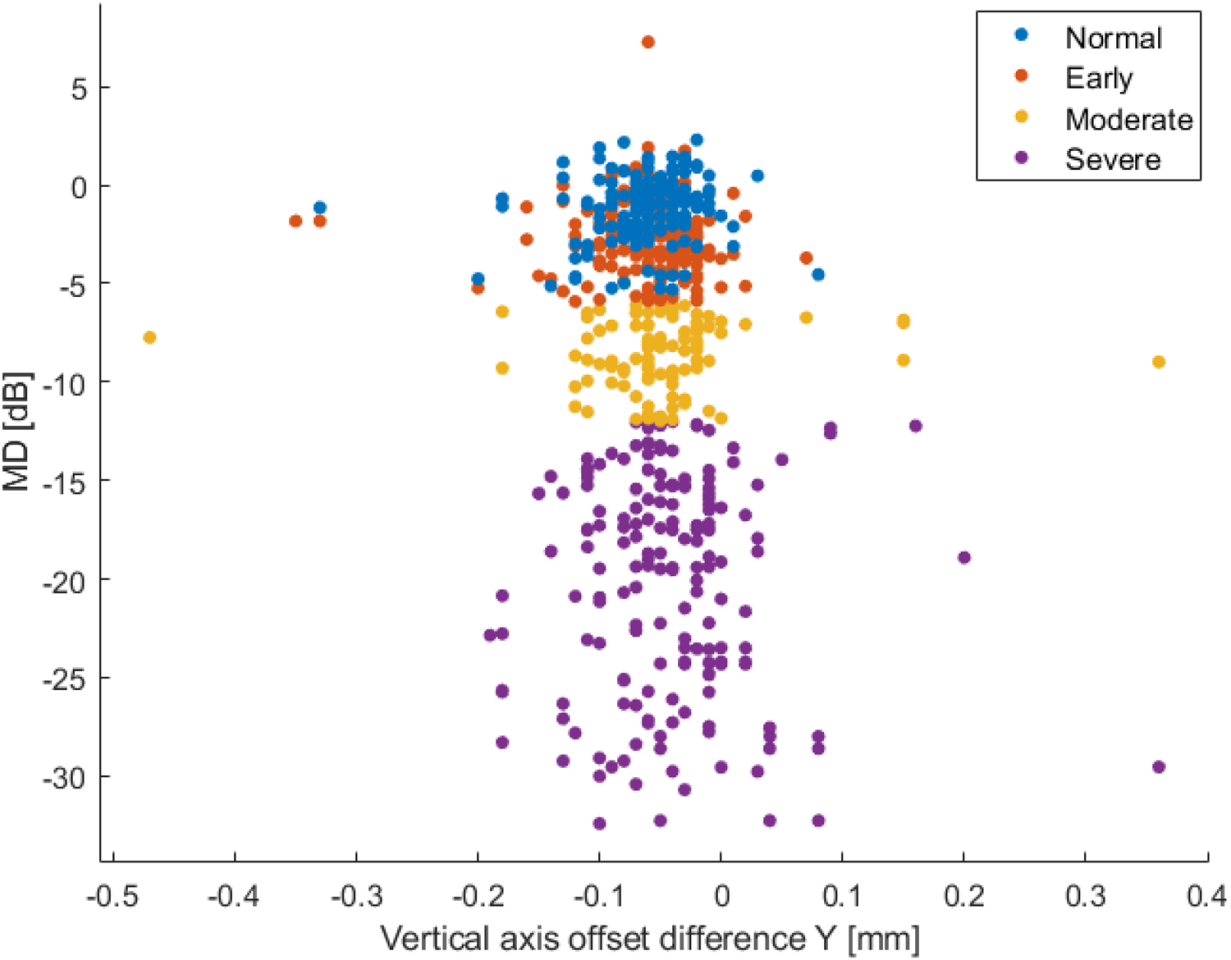
Classes distribution of vertical offset difference regard to vertical axis and the mean deviation (MD).

The classes distribution of corneal eccentricity in 4 mm region and the MD is presented in Fig. 7. Figure 8 shows the classes distribution of vertical offset difference versus mean deviation (MD). The two represented cornel parameters are the most important ones when predicting the visual field damage, and we can observe that the classes are very well separated conforming our feature selection approach previously presented.

Table 5 summarizes the corneal characteristics by VF impairment class allocation. Table presents the calculated *p* values. From the obtained results the *p* values are below 0.05, which suggest that the difference between the normal and abnormal VF classes for the identified corneal parameter is significant.

**Table 5:** Corneal characteristics analysis by VF impairment class allocation.

Selected corneal parameter	Normal VF		Abnormal VF		*p* value
	Mean ± standard deviation (SD)	Range	Mean ± Standard Deviation (SD)	Range	
Corneal eccentricity (4 mm region)	− 0.1 ± 0.67	− 0.98 to 2.32	− 0.1 ± 0.74	− 0.97 to 2.52	0.03
Vertical axis offset difference Y axis (mm)	− 0.06 ± 0.04	− 0.33 to 0.08	− 0.05 ± 0.61	− 0.47 to 0.36	0.02
Base curve (BC) axis (mm)	0.01 ± 0.02	− 0.05 – 0.15	0.01 ± 0.03	− 0.09 to 0.31	0.02
Elevation Anterior Index of the BFS Y axis (mm)	0.01 ± 0.02	− 0.05 to 0.14	0.01 ± 0.04	− 0.08 to 0.42	0.04
Pupillary offset (mm)	0.11 ± 0.11	0.01–0.85	0.13 ± 0.107	0.01–0.87	< 0.001

Values are presented as mean ± standard deviation.

## Discussions

The current study demonstrates that visual field damage and severity level can be identified from the corneal shape, thickness, and elevation parameters with reasonable accuracy. Although it has been shown that CCT and CH are predictors of glaucoma development and its progression, there is no report in the literature on the association between glaucoma and detailed corneal shape, thickness, and elevation. Our finding suggests that corneal shape, thickness, and elevation may predict visual field damage and may represent additional glaucoma risk factors.

Glaucoma etiology remains mostly unknown and several factors such as age, ethnicity, IOP, and CCT may interact in a complex way in disease development^28,45^. Presently, IOP remains the only modifiable risk factor, however, subjects with elevated IOP or even other risk factors may not develop glaucoma. Likewise, subjects with low or well-controlled IOP may develop glaucoma^46^. Therefore, other known or unknown factors—besides IOP—likely influence glaucoma development and progression. We show that visual field damage can be detected from corneal parameters which may suggest an association between corneal structural parameters and glaucomatous visual field damage.

Our proposed machine learning approach achieved an AUC of 0.83 in detecting visual field damage from corneal parameters. While this level of accuracy may not be ideal for some tasks, detecting visual field damage in the posterior segment of the eye from corneal structural parameters in the anterior segment of the eye, is critical as it shows an association between corneal parameters and glaucomatous visual field loss. Machine learning models not only detected visual field damage from corneal parameters with good accuracy but also detected different glaucoma severity levels from corneal parameters. Machine learning achieved an average AUC of 0.74 in detecting normal, early, moderate, and severe visual field loss from corneal parameters. Detecting different glaucomatous visual field severity levels is consistent with detecting visual field damage which may suggest that part of glaucoma etiology may be explained by corneal shape, elevation, and thickness.

Previous studies have identified CCT and CH as strong predictors of glaucoma development and progression^3,47,48^. We identified a total of 12 topography, pachymetry, elevation, and keratometry characteristics of cornea predicted visual field damage as well as visual field severity level with an AUC of 0.74. The majority of these parameters belong to corneal topography (corneal-map mode) including the height of the corneal anterior eccentricity at 4, 12, and 15 mm zones. Eccentricity reflects the amount of corneal flattening from the center to the periphery. Other topography parameters were the elevation anterior index of the best fit surfaces (BFS). The best-fit spheres are computed for the anterior and posterior corneal surfaces independently, and the differences between these two fitted surfaces are plotted as elevation maps. Best fit square provides a useful metric in clinic for most patients. It is worth mentioning that findings confirm our previous statistical analysis^49^ that suggested several corneal shape and elevation parameters are strongly associated with visual field severity level.

In real clinical settings, fitting a BFS to the central 8 to 9-mm zone is typically feasible, as this provides adequate data points to obtain maps without requirement of extrapolation. An eye with normal cornea has an aspherical prolate surface in the central 8- to 9-mm zone and BFS in this region allows for identification of subtle changes in both ectatic disorders and astigmatism. Out of these 12 corneal parameters, seven belong to shape (topography), two parameters belong to thickness (pachymetry), two parameters belong to elevation, and one parameter corresponds to keratometry (aberration) using topo-pachy-map scan protocol. While the association between the thickness of central cornea and glaucoma has been long established, the role of additional thickness parameters may be explained accordingly. However, the roles of corneal shape, elevation, and keratometry in glaucoma status are novel and worth further investigation.

In our previous studies, we identified corneal parameters that predicted keratoconus severity and the risk of future keratoplasty^50–52^. We showed that keratoconus severity can be detected from several corneal parameters including keratometry and eccentricity with a high accuracy. In this study also found eccentricity and keratometry, along with several other corneal parameters, that can detect glaucomatous visual field damage. While the association between myopia and glaucoma is well studied^53,54^, follow-up studies may be merited to further examine the association between keratoconus and glaucoma, as our studies suggest that several corneal parameters such as spherical aberration coefficients exist that can predict both conditions.

While several studies have proposed statistical and machine learning models to estimate visual field severity level from retinal OCT data, our study provides a pilot study suggesting there is an association between visual field severity level and corneal structure. While predicting retinal functional data from retinal structural data may seem not too challenging, predicting visual field severity from corneal structure seem both technically and clinically interesting.

Although we investigated several machine learning models and assessed the ability to detect both visual field damage (abnormality) and visual field severity levels, our study has several limitations that can be addressed in future studies. We collected corneal parameters and visual field data from eyes and allowed a 1-year time difference between anterior segment OCT and visual field test exams, which may seem too long for glaucoma. Limiting corneal measurement and visual field test intervals to 6 months led to a small subset with 381 eyes which was not ideal for machine learning analysis. However, there are glaucoma related structure–function studies with an allowance of a 1-year interval between structure and function tests^55^. Additionally, there are several studies in the literature that have used multi-modal analysis based on 1 year interval between the capturing data of the two data modalities^56^.

Another limitation is that we collected corneal parameters using OCT technology in CASIA instruments (mostly used instrument in Japan); however, Schimpflug imaging, as used in the Pentacam instrument, is another widely used technology for measuring corneal parameters. Other studies with Pentacam data are desirable to validate findings. However, Chan et al.^57^ reported that CASIA provided a better repeatability in measuring corneal thickness and posterior elevation with important clinical implications in the diagnosis and monitoring of disease progression in keratoconus. Moreover, several other studies also showed that that CASIA imaging parameters are highly repeatable and reproducible^58,59^.

The other limitation is that the cohort is from Japan; therefore, other datasets from other ethnicities/countries may be required to generalize our findings. Finally, while rare, our subset may include a few eyes with corneal opacity, corneal erosion, dry eye, refractive surgery, lens status, or even other retinal conditions, however, we utilized large number of eyes that mitigate the effect of such possibilities on results. Visual field test is subjective and typically takes a long time to complete which is challenging for older patients. Additionally, it is well known that visual field tests are variable, particularly in the late stage of the glaucoma^60,61^. These facts make our study even more critical as estimating visual field severity levels from corneal imaging data may augment or even replace subjective and tedious visual field testing with objective and more rapid corneal imaging. However, this is a feasibility study that requires further investigation and validation using independent datasets.

In conclusion, our study showed an association between visual field damage and corneal shape. Since glaucoma can be asymptomatic, particularly at the early stages of the disease, its detection before significant vision loss is critical^62^. As such, methods for detecting glaucoma could significantly impact public health. In the current study, we have proposed machine learning models for detecting visual field damage from corneal parameters. Essentially, rather than using glaucoma classic risk factors or routinely used visual field or imaging modalities in clinical settings, corneal parameters may be used to predict glaucomatous visual field defect. Further studies are warranted to identify whether there is a link between corneal shape in patients with keratoconus and visual field damage in glaucoma or where keratoconus could be a risk factor for glaucoma development and future visual field loss and optic nerve damage. Moreover, follow-up studies could identify the association between corneal structural parameters and major glaucoma risk factors such as age, IOP and CCT.
